# 高速逆流色谱法制备罗汉果根中葫芦素类化合物

**DOI:** 10.3724/SP.J.1123.2021.07010

**Published:** 2022-04-08

**Authors:** Jiayi SUN, Jiaqi SUN, Heping LI, Xiaojie YAN, Dianpeng LI, Fenglai LU

**Affiliations:** 1.桂林理工大学化学与生物工程学院, 广西 桂林 541006; 1. College of Chemistry and Bioengineering, Guilin University of Technology, Guilin 541006, China; 2.广西壮族自治区中国科学院 广西植物研究所, 广西植物功能物质研究与利用重点实验室, 广西 桂林 541006; 2. Guangxi Key Laboratory of Functional Phytochemicals Research and Utilization, Guangxi Institute of Botany, Chinese Academy of Sciences, Guangxi Zhuang Autonomous Region, Guilin 541006, China

**Keywords:** 高速逆流色谱, 高效液相色谱, 制备, 葫芦素, 罗汉果根, high speed countercurrent chromatography (HSCCC), high performance liquid chromatography (HPLC), cucurbitacin, preparation, *Siraitia grosvenorii* roots

## Abstract

葫芦素作为四环三萜类化合物广泛存在于葫芦科植物中,但其含量较低、结构相似,采用常规的柱层析分离法较难得到大量、高纯度的单体化合物,导致其活性的研究与应用受到限制。研究采用高速逆流色谱法(HSCCC),建立了一种从罗汉果根提取物中制备葫芦素类化合物的方法。罗汉果根乙醇提取物经HPD-100大孔树脂、MCI、RP-C18柱层析分离后获得葫芦素粗品。通过基于薄层色谱的溶剂选择法(GUESS)对液-液萃取与高速逆流色谱的溶剂体系进行快速筛选,确定采用正己烷-乙酸乙酯-甲醇-水(3:7:3:7, v/v/v/v)萃取葫芦素粗品,有机层萃取物再以正己烷-乙酸乙酯-甲醇-水(4:6:5:5, v/v/v/v)进行HSCCC分离,以上相作为固定相,下相作为流动相,流速2.0 mL/min,主机转速860 r/min,进样量280 mg,一次分离即可获得5种葫芦素类化合物,经高效液相色谱检测其纯度分别为97.0%、95.4%、96.3%、91.6%和95.3%,并通过核磁共振技术、高分辨质谱技术及文献资料对比对各化合物的结构进行鉴定。经鉴定,5种化合物分别为葫芦素Q1、23,24-二氢葫芦素F-25-乙酸酯、葫芦素B、23,24-二氢葫芦素B和二氢异葫芦素B-25乙酸酯。该方法操作简单、快速,分离效果好,可作为规模化制备葫芦素类化合物的一种新方法。

## Abstract (en)

*Siraitia grosvenorii* (Swingle) C. Jeffrey, belonging to the family Cucurbitaceae, is a natural sweetener. The roots of this plant are used in folk medicine for the treatment of rheumatoid arthritis. Cucurbitacins play an important role in the resistance of this plant to insects and adversity, and have anti-inflammatory, anti-tumor, and other biological activities. They usually exist as a variety of similar structures in Cucurbitaceae plants. Separation of a large amount of high-purity monomer compounds by the conventional separation method based on column chromatography is difficult, which limits the research and application of their activities. Therefore, we chose a new method for this separation. High-speed countercurrent chromatography (HSCCC) is a liquid-liquid chromatographic technique characterized by high recovery and reproducibility, and is considered a very effective method for the separation of natural compounds present in various plant extracts. An appropriate solvent system is the key for efficient separation, but its selection is tedious, which hampers the wider implementation of HSCCC in chemical research involving preparative separations. In this study, based on the general estimation strategy by using the TLC solvent system (GUESS), the corresponding relationship between the partition distribution coefficient (*K* value) and the TLC retention factor (*R*_f_ value) of the compounds was established by the partition experiment. The *R*_f_ value and separation coefficient *α* were calculated using the water-saturated organic phase as the expansion agent, which could minimize the number of countercurrent separation experiments required in solvent system selection. In this study, HSCCC was used to establish an efficient method for the extraction of cucurbitacins from the root extract of *Siraitia grosvenorii*. A fraction rich in cucurbitacins was obtained from the ethanol extract of *Siraitia grosvenorii* roots after separation by column chromatography on HPD-100, MCI, and C18 columns. Six types of solvent systems with different compositions were investigated using the GUESS method. The results showed that employing the solvent system of *n*-hexane-ethyl acetate-methanol-water (3:7:3:7, v/v/v/v) to partition the cucurbitacin fraction could remove a large number of impurities. The components retained in the upper phase in the partition experiment were subsequently purified by HSCCC. The favorable solvent system for HSCCC was *n*-hexane-ethyl acetate-methanol-water (4:6:5:5, v/v/v/v), while the upper and lower phases were selected as the stationary and mobile phases, respectively, with a flow rate of 2.0 mL/min, a rotation speed of 860 r/min, and an injected sample weight of 280 mg. Five cucurbitacin compounds were obtained by one-time separation. The weights of the five compounds were 14.73, 8.82, 30.74, 5.03, and 3.81 mg. The purities of these compounds were 97.0%, 95.4%, 96.3%, 91.6%, and 95.3%, respectively. Their structures were identified as cucurbitacin Q1, 23,24-dihydrocucurbitacin F-25-acetate, cucurbitacin B, 23,24-dihydrocucurbitacin B, and dihydroisocucurbitacin B-25-acetate by^1^H-NMR and ^13^C-NMR spectroscopies, along with comparison with the literature. This study demonstrated how GUESS guidance accelerates the selection of HSCCC solvent systems, simplifies the workflow, and it provides an efficient preparative method for the separation of chemical constituents from the *Siraitia grosvenorii* roots, which can also be used as a new method for the large-scale preparation of cucurbitacin compounds.

葫芦科植物罗汉果(*Siraitia grosvenorii* (Swingle) C. Jeffrey)作为一种具有中国特色的药用和甜料植物,是国家卫生部首批公布的药食同源中药材,其果实具有良好的药用价值,同时具有很强的经济价值^[1]^。罗汉果除被用作药材和饮片外,还被制成颗粒、糖浆、胶囊、片剂、酊水剂、散剂等6大剂型共80余种中成药,涉及数十家药企,产值达百亿。目前罗汉果的应用及研究都集中在罗汉果的果实上,而每年挖出的根通常被丢弃,造成很大的资源浪费。《中华本草》和《中国民族药志》等均记载了罗汉果根的主治功能,包括“利湿止泻;舒筋。主腹泻;舌胖;脑膜炎后遗症”,是桂林地区民间一种标志性药材^[2]^。据目前的文献^[3,4,5,6]^报道,罗汉果根中的化学成分基本为葫芦烷型四环三萜类化合物。课题组前期^[7]^从罗汉果根中分离得到葫芦素B等葫芦素类化合物,该类化合物是葫芦科植物的代表性成分,具有显著的抗肿瘤^[8]^、抗炎^[9]^、保肝^[10]^和提高机体免疫力^[11]^等功效,这类成分很有可能是罗汉果根中的主要活性成分,因此非常有必要进一步明确葫芦素类化合物的结构,同时获得单体化合物,为今后药理活性的进一步研究以及质量控制提供对照品。

目前,罗汉果根化学成分的研究主要采用硅胶柱层析等手段,而硅胶对于皂苷的吸附性较强,死吸附严重,不利于样品的回收。高速逆流色谱(HSCCC)作为一种新型液-液色谱分离技术,无固体载体,具有样品无损失、无污染、高效快速的优点,非常适合植物中天然产物活性成分的分离^[12,13,14,15]^。

基于薄层色谱溶剂选择法(GUESS)是由Friesen和Pauli教授^[16]^2005年发展创立的快速筛选HSCCC溶剂体系的方法。该方法是在硅胶板上采用平衡好的两相溶剂中的有机层作为展开剂对样品检测,以样品在薄层色谱板(TLC)上的比移值(*R*_f_)来判断溶剂体系的合理性。与传统的溶剂选择方法相比,该方法避免了分配系数*K*值的测定及其相关的大量实验,更简便易行。

本文通过GUESS选择溶剂系统并利用液-液萃取与HSCCC结合的方法对罗汉果根中葫芦素类化合物进行分离纯化,缩短了溶剂体系的选择时间,提高了分离效率,且具有很好的分离效果,值得推广应用。

## 1 实验部分

### 1.1 仪器、试剂与材料

液相色谱仪(LC-2030C,岛津企业管理(中国)有限公司);超声波清洗机(SB-5200D,宁波新芝生物科技股份有限公司);电子分析天平(XS225A-SCS,普利赛斯国际贸易(上海)有限公司);高速逆流色谱仪TBE-300C、低温恒温槽DC-0506、恒温泵TBP-5002(上海同田生物技术有限公司);自动接收仪(CHF161RA,日本Advantec公司);真空离心浓缩仪(miVac,英国Gene Vac公司);超导核磁共振波谱仪(Brucker Avance 500MHz,瑞典Brucker公司);超高效液相色谱-电喷雾离子阱飞行时间质谱联用仪(LCMS-IT-TOF,日本Shimadzu公司)。

HPD-100大孔树脂(赛普锐斯(北京)科技有限公司); RP-C18层析柱(日本Fuji Silysia Chemical Ltd公司); MCI层析柱(日本三菱化学);硅胶薄层板F_254_(厚度为0.2 mm,德国默克公司);正己烷(分析纯,西陇科学股份有限公司);乙酸乙酯(分析纯,广东光华科技股份有限公司);甲醇(分析纯,成都市科隆化学品有限公司);饮用纯净水(娃哈哈股份有限公司);乙腈(色谱纯,美国Fisher Scientific公司);氘代吡啶(C_5_D_5_N,美国CIL公司,包含0.03%四甲基硅烷(TMS))。

罗汉果根采自广西桂林永福县龙江乡,经广西壮族自治区中国科学院广西植物研究所鉴定为葫芦科属植物罗汉果的块根。

### 1.2 实验方法

1.2.1 提取及样品预处理

罗汉果块根洗净阴干后粉碎,得到罗汉果根粉末2 kg,用70%(体积分数,下同)乙醇水溶液浸提,将提取液浓缩后经HPD-100大孔树脂柱层析,依次用纯水和20%、40%、60%、80%、100%乙醇水溶液梯度洗脱。将60%乙醇水溶液洗脱部分经MCI柱层析,依次用纯水和不同体积分数的甲醇水溶液梯度洗脱(10%~100%,每10%为一个梯度),收集70%~80%甲醇水溶液,将洗脱液经RP-C18柱层析分离后,收集富含葫芦素类化合物的流分,获得1 g葫芦素粗品。以正己烷-乙酸乙酯-甲醇-水(3:7:3:7, v/v/v/v)对葫芦素粗品进行液-液萃取,对有机相萃取物进行HSCCC分离制备。

1.2.2 高速逆流色谱分离条件

称取280 mg有机相萃取物作为待分离样品。选择正己烷-乙酸乙酯-甲醇-水(4:6:5:5, v/v/v/v)作为溶剂体系,将两相溶剂系统在分液漏斗中配制,充分振荡后分为上下两相,上相(有机层)作为固定相,下相(水层)作为流动相。将分层后的两相分别用超声波清洗机进行超声脱气20 min,分别取上下相溶剂各5 mL溶解待分离样品。以流速30 mL/min将固定相注入螺旋管内,调主机转速为860 r/min,待固定相充满整个螺旋管后设流速为2 mL/min,泵入流动相平衡。待流动相从主机出口处流出时,注入溶解后的样品。温度设定为30 ℃,检测波长254 nm。

1.2.3 色谱条件和质谱条件

色谱条件:采用分析型Agilent Poroshell120 SB-C18色谱柱(150 mm×4.6 mm, 4 μm,美国Agilent公司);柱温30 ℃;以乙腈(A)和纯水(B)作为流动相,梯度洗脱程序为0~35 min, 20%A~50%A;流速0.8 mL/min;检测波长200 nm。

质谱条件:电离源为电喷雾电离源,负离子模式(ESI^-^);扫描范围:*m/z* 200~1000;喷雾室电压为-3.5 kV;雾化气流速为1.50 L/min;曲型脱溶剂管温度:200 ℃;检测器电压:1.70 kV;碰撞诱导解离能量为20 eV。

1.2.4 纯度测定及结构鉴定

将高速逆流色谱制备得到的样品旋干后称重,并用高效液相色谱进行分析检测,用面积归一化法测定纯度。将样品用氘代吡啶试剂溶解进行核磁共振和质谱分析,鉴定其化学结构。

## 2 结果与讨论

### 2.1 提取条件的选择

将2 kg罗汉果根粉末经70%乙醇水溶液浸提,提取液浓缩后以HPD-100大孔树脂、MCI柱、RP-C18柱层析分离,得到葫芦素粗品。以正己烷-乙酸乙酯-甲醇-水(3:7:3:7, v/v/v/v)溶剂体系中分层后的有机相作为展开剂对组分进行TLC分析,用硫酸显色剂显色后发现样品中一部分成分的*R*_f_值较高,而另一部分的*R*_f_值则几乎为零,表明样品中存在极性相差较大的成分,采用这个体系萃取样品,能将样品中不同极性的物质分配在两相中,可极大地减少样品的复杂性。采用1.2.3节的方法检测,证实了葫芦素粗品萃取前(见图1a)成分比较复杂,而萃取后的HPLC图谱显示有机层和水层中的化学成分显著不同(见图1b和图1c)。该结果表明采用正己烷-乙酸乙酯-甲醇-水四元体系对样品进行萃取预处理可以除去大部分杂质^[17]^。为进一步纯化葫芦素类成分,采用HSCCC对上相萃取物进行分离制备。

**图1:**
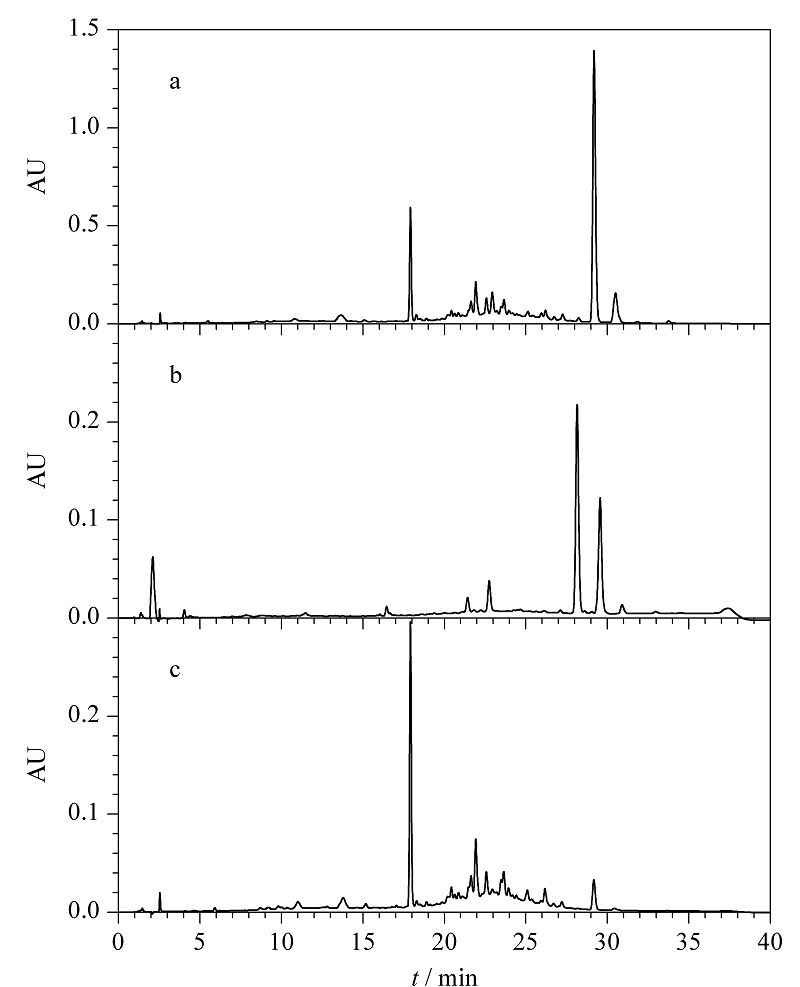
葫芦素粗品萃取前后的HPLC色谱图

### 2.2 HSCCC溶剂体系的选择

HSCCC能否成功分离化合物取决于溶剂系统的选择。薄层色谱常应用于逆流色谱分离,对分离得到的流分进行化学成分分析,分析物在薄层硅胶板上的分布与其极性具有很强的相关性。根据这一原理发展出来的GUESS法中,化合物在溶剂体系中的*K*与其*R*_f_值之间有一定对应关系^[16]^。Liu等^[18]^通过43种天然产物对*R*_f_与*K*之间关系的理论模型进行了验证,结果均表明,*R*_f_的最佳取值范围应以0.5为中心;验证了当0.4<*K*<2.5时,对应0.29<*R*_f_<0.71;其中*R*_f_=0.5时,*K*约等于1。因此当以水饱和的有机层作为展开剂,当待分离样品中主要化合物的*R*_f_值为0.29~0.71时,可认为所选择的溶剂系统具有较好的分离效果。同时可根据*R*_f_值选择固定相与流动相,若*R*_f_值<0.5,说明分离组分极性较大,可选用溶剂体系中极性较强的一相作为流动相^[12]^。根据目标化合物的极性及相关文献^[19,20]^,选择正己烷-乙酸乙酯-甲醇-水作为溶剂体系。

本文依据样品的极性特征,在不需要计算目标化合物分配系数的基础上,采用TLC考察了6种不同体系配比下各目标化合物的*R*_f_值及分离系数*α*(见表1)。结果表明,在不同正己烷-乙酸乙酯-甲醇-水比例的体系中,各化合物的*R*_f_值及*α*有明显不同,其在正己烷-乙酸乙酯-甲醇-水(4:6:5:5, v/v/v/v)与正己烷-乙酸乙酯-甲醇-水(4:6:4:6, v/v/v/v)体系中的范围比其他体系更合理。虽然在正己烷-乙酸乙酯-甲醇-水(4:6:5:5, v/v/v/v)中,化合物1的*R*_f_值<0.29,但化合物3的*R*_f_值最接近0.5,且各化合物的*α*均大于正己烷-乙酸乙酯-甲醇-水(4:6:4:6, v/v/v/v)体系。因此,正己烷-乙酸乙酯-甲醇-水(4:6:5:5, v/v/v/v)溶剂系统被认为适合用于HSCCC分离。

**表1:** 化合物在不同溶剂体系中的*R*_f_和*α*

H:E:M:W	R_f_					α_1_	α_2_	α_3_	α_4_
	No. 1	No. 2	No. 3	No. 4	No. 5				
5:5:6:4	0.04	0.10	0.20	0.26	0.28	2.50	2.00	1.30	1.08
5:5:5:5	0.16	0.24	0.42	0.50	0.54	1.50	1.75	1.19	1.08
4:6:6:4	0.18	0.24	0.42	0.48	0.54	1.33	1.75	1.14	1.13
4:6:5:5	0.22	0.30	0.49	0.60	0.64	1.36	1.63	1.22	1.07
4:6:4:6	0.31	0.38	0.58	0.66	0.70	1.23	1.53	1.14	1.06
3:7:3:7	0.58	0.64	0.80	0.84	0.88	1.10	1.25	1.05	1.05

H:E:M:W: *n*-hexane-ethyl acetate-methanol-water, v/v/v/v; *α*_1_=*R*_f2_/*R*_f1_, *α*_2_=*R*_f3_/*R*_f2_, *α*_3_=*R*_f4_/*R*_f3_, *α*_4_=*R*_f5_/*R*_f4_.

### 2.3 HSCCC分离纯化的结果

用正己烷-乙酸乙酯-甲醇-水(4:6:5:5, v/v/v/v)体系分离样品,上层溶剂(有机层)作为固定相,下层溶剂(水层)作为流动相,HSCCC的分离情况见图2。结合流分的TLC检测结果,将HSCCC图谱划分成5个部分。采用1.2.3节的方法分析并用峰面积归一法计算各化合物纯度,得到的5个化合物(峰1~5)的纯度分别为97.0%、95.4%、96.3%、91.6%和95.3%(HPLC色谱图见图3);对5个化合物减压蒸干后进行称重,得到重量分别为14.73、8.82、30.74、5.03和3.81 mg。

**图2:**
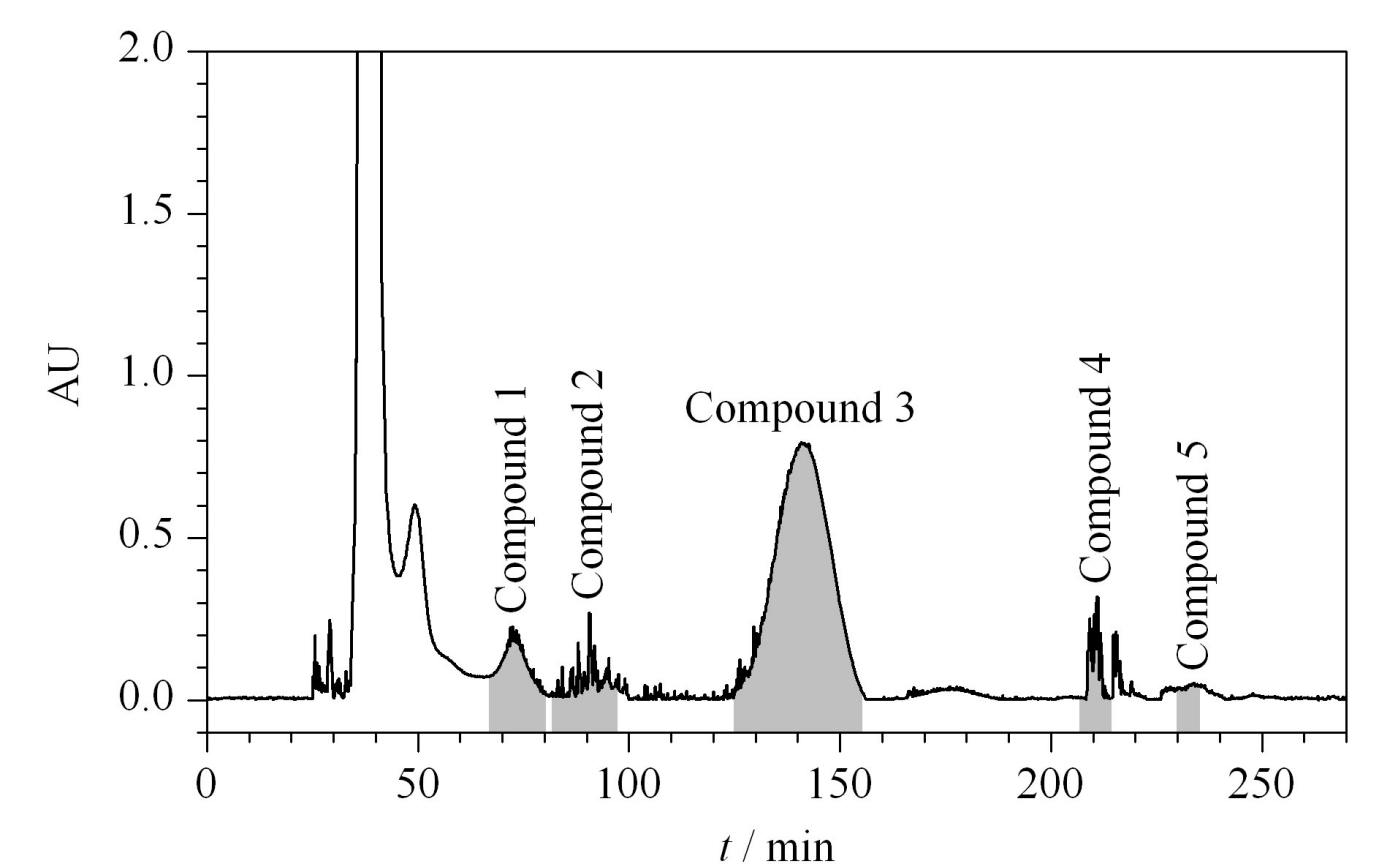
罗汉果根葫芦素类化合物的高速逆流色谱图

**图3:**
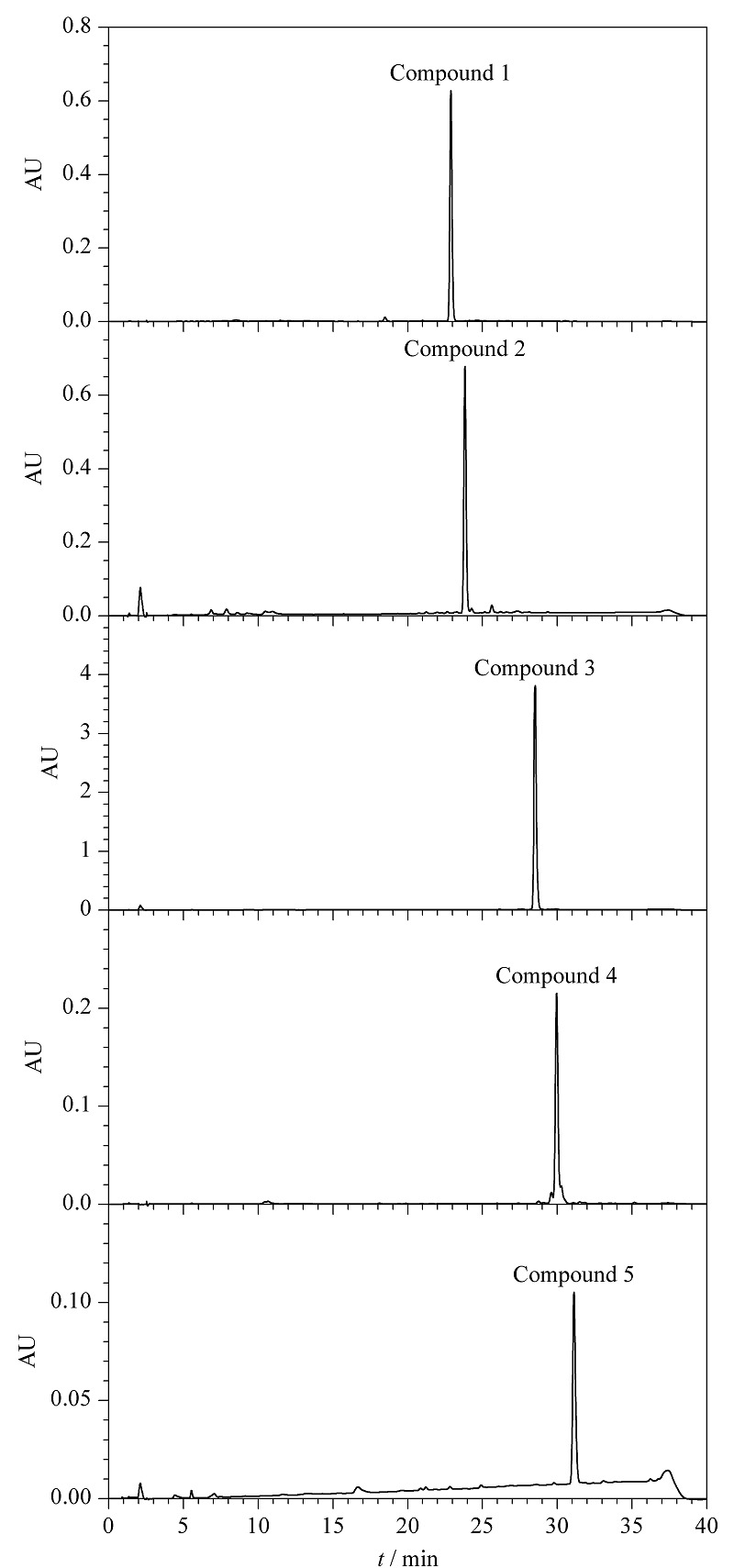
化合物1~5的HPLC色谱图

### 2.4 化合物的结构鉴定

将HSCCC分离得到的化合物进行^1^H-NMR、^13^C-NMR及质谱分析测定,确定其化学结构,结果如下。

化合物1:白色针状结晶;[M+HCOO]^-^ (*m/z*): 605.33,相对分子质量为560.33,推测分子式为C_32_H_48_O_8_;^1^H-NMR (500 MHz, C_5_D_5_N) *δ*: 7.41 (d, *J*=15.8 Hz, H-23), 7.38 (d, *J*=15.8 Hz, H-24), 6.33 (d, *J*=4.83 Hz, 3-OH), 6.19 (s, 16-OH), 5.74 (d, *J*=4.83 Hz, H-6), 4.11 (ddd, *J*=11.4, 9.0, 4.1 Hz, H-2), 3.45 (d, *J*=9.1 Hz, H-3), 3.33 (d, *J*=14.5 Hz, H-12), 3.05 (d, *J*=7.0 Hz, H-17), 2.87 (d, *J*=14.5 Hz, H-12), 2.73 (d, *J*=13.2 Hz, H-10), 2.45 (ddd, J=12.3, 3.08, 4.0 Hz, H-1), 2.35 (m), 1.96 (d, *J*=7.68 Hz, H-8), 1.91 (s, 25-OAc), 1.76 (d, *J*=12.9 Hz, H-15), 1.72 (s), 1.58 (s), 1.57 (s), 1.53 (s), 1.49 (s), 1.31 (s), 1.26 (s), 1.23 (s);^13^C-NMR (126 MHz, C_5_D_5_N) *δ*: 213.76 (C-11), 204.86 (C-22), 170.31 (COCH_3_), 149.90 (C-24), 142.94 (C-5), 123.00 (C-23), 119.22 (C-6), 81.91 (C-20), 80.33 (C-3), 80.26 (C-25), 71.50 (C-16), 71.27 (C-2), 60.14 (C-17), 51.60 (C-14), 49.55 (C-12), 49.36 (C-13), 49.08 (C-9), 46.86 (C-15), 43.72 (C-10), 43.29 (C-4), 35.14 (C-1), 34.92 (C-8), 27.06 (C-28), 26.68 (C-27), 25.96 (C-21), 25.94 (C-29), 24.68 (C-21), 22.91 (C-26), 22.21 (COCH_3_), 21.03 (C-19), 20.89 (C-30), 19.66 (C-18)。以上数据与文献^[21]^报道基本一致,鉴定该化合物为葫芦素Q1,化学结构式如图4a所示。

**图4:**
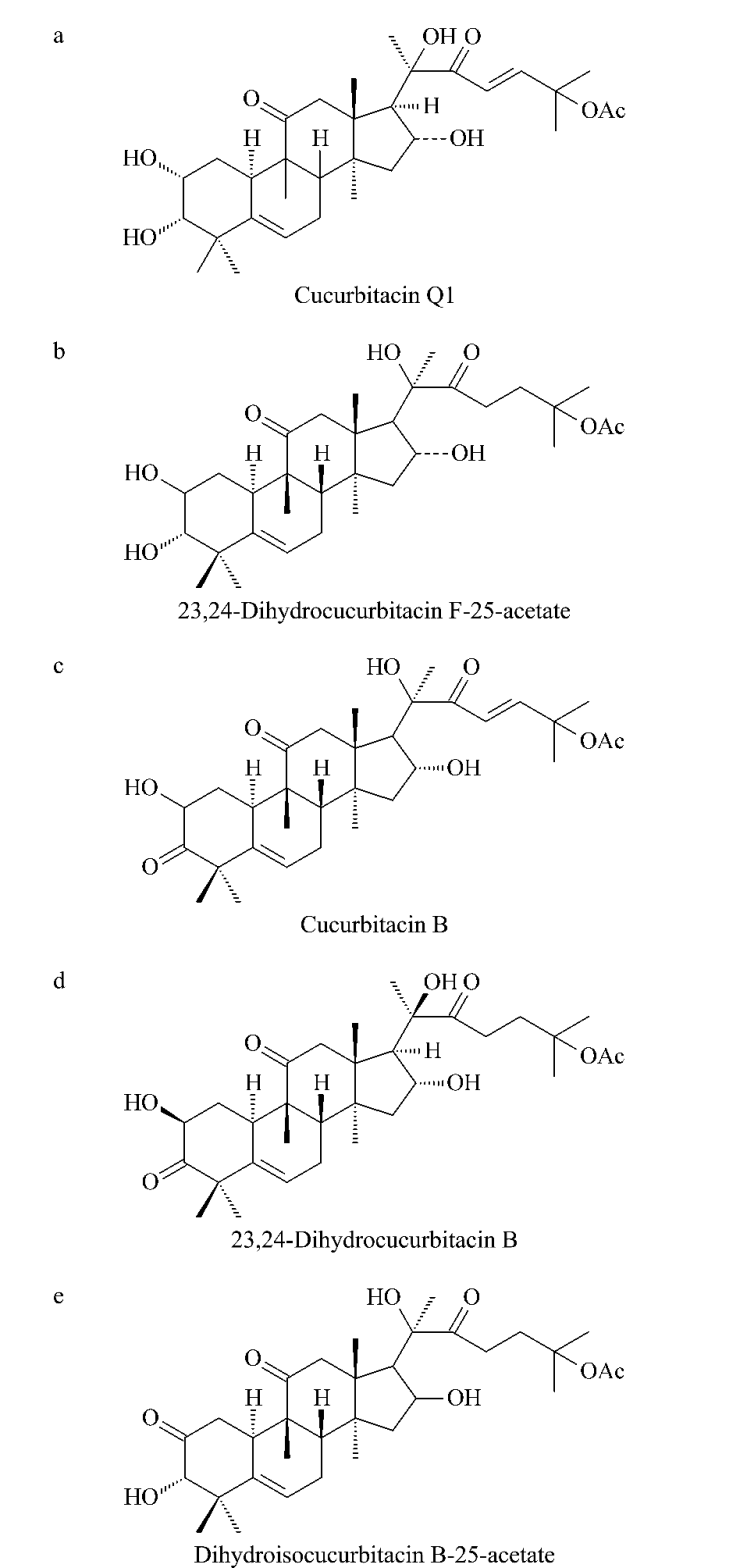
化合物1~5的结构式

化合物2:白色针状结晶;[M+HCOO]^-^ (*m/z*): 607.35,相对分子质量为562.35,推测分子式为C_32_H_50_O_8_;^1^H-NMR (500 MHz, C_5_D_5_N) *δ*: 6.50 (1H, s), 5.96 (2H, s), 5.75 (1H, d, *J*=6.3 Hz), 4.96 (2H, s), 4.13 (1H, s), 3.46 (1H, d, *J*=9.1 Hz), 3.34 (2H, dd, *J*=16.6, 13.3 Hz), 3.14 (1H, dd, *J*=11.0, 5.1 Hz), 2.99 (1H, d, *J*=7.1 Hz), 2.85 (1H, d, *J*=14.5 Hz), 2.74 (1H, d, *J*=13.1 Hz), 2.47 (2H, dt, *J*=10.6, 5.6 Hz), 2.36 (2H, m), 1.92 (3H, s), 1.73 (1H, d, *J*=13.0 Hz), 1.63 (2H, s), 1.58 (2H, s), 1.56 (3H, s), 1.51 (8H, d, *J*=10.6 Hz), 1.32 (3H, s), 1.26 (6H, d, *J*=11.1 Hz);^13^C-NMR (126 MHz, C_5_D_5_N) *δ*: 215.61 (C-22), 213.64 (C-11), 170.62 (COCH_3_), 142.93 (C-5), 119.21 (C-6), 82.09 (C-20), 81.90 (C-3), 80.66 (C-25), 71.49 (C-2), 70.88 (C-16), 59.47 (C-17), 51.56 (C-14), 49.75 (C-12), 49.32 (C-13), 49.20 (C-9), 46.89 (C-15), 43.60 (C-10), 43.29 (C-4), 35.88 (C-24), 35.15 (C-1), 34.91 (C-8), 32.66 (C-23), 26.53 (C-28), 26.43 (C-26), 25.99 (C-27), 25.95 (C-21), 24.64 (C-7), 22.92 (COCH_3_), 22.67 (C-29), 20.89 (C-18), 20.84 (C-19), 19.65 (C-30)。以上数据与文献^[22]^报道基本一致,鉴定该化合物为23,24-二氢葫芦素F-25-乙酸酯,化学结构如图4b所示。

化合物3:淡黄色针状结晶;[M+HCOO]^-^ (*m/z*): 603.32,相对分子质量为558.32,推测分子式为C_32_H_46_O_8_;^1^H-NMR (500 MHz, C_5_D_5_N) *δ*: 7.41 (1H, m), 7.38 (1H, m), 1.92 (3H, s, COCH_3_), 1.73 (3H, s, H-21), 1.60 (3H, s, H-2), 1.57 (3H, s, H-2), 1.54 (3H, s, H-26), 1.46 (3H, s, H-28), 1.30 (3H, s, H-29), 1.22 (3H, s, H-19), 1.14 (3H, s, H-1);^13^C-NMR (126 MHz, C_5_D_5_N) *δ*: 213.6 (C-3), 213.3 (C-11), 204.8 (C-22), 150.4 (C-24), 141.7 (C-5), 122.9 (C-23), 120.6 (C-6), 80.2 (C-25), 80.1 (C-20), 72.8 (C-2), 71.1 (C-16), 70.2 (COCH_3_), 60.3 (C-17), 51.4 (C-14), 51.3 (C-4), 49.5 (C-12), 49.2 (C-9), 49.0 (C-13), 46.7 (C-15), 43.3 (C-8), 37.3 (C-1), 34.6 (C-10), 29.8 (C-28), 26.9 (C-27), 26.6 (C-26), 26.0 (C-21), 24.6 (C-7), 22.2 (COCH_3_), 22.1 (C-29), 21.0 (C-18), 20.4 (C-19), 19.3 (C-30)。以上数据与文献^[23]^报道基本一致,鉴定该化合物为葫芦素B,化学结构式如图4c所示。

化合物4:白色针状结晶;[M+HCOO]^-^ (*m/z*): 605.33,相对分子质量为560.33,推测分子式为C_32_H_48_O_8_;^1^H-NMR (500 MHz, C_5_D_5_N) *δ*: 6.54 (1H, d, *J*=4.6 Hz), 6.39 (1H, d, *J*=4.4 Hz), 6.04 (1H, s), 5.71 (1H, dd, *J*=5.7, 2.4 Hz), 3.35 (2H, m), 3.10 (2H, m), 2.98 (1H, d, *J*=7.1 Hz), 2.90 (1H, d, *J*=14.5 Hz), 2.70 (1H, ddd, *J*=12.3, 5.8, 3.5 Hz), 2.47 (1H, ddd, *J*=15.3, 11.0, 4.6 Hz), 2.35 (2H, m), 1.96 (2H, d, *J*=7.5 Hz), 1.93 (3H, s, COCH_3_), 1.71 (2H, m), 1.63 (3H, s, H-30), 1.58 (3H, s, H-21), 1.52 (3H, s, H-27), 1.50 (3H, s, H-26), 1.46 (3H, s, H-28), 1.32 (3H, s, H-29), 1.24 (3H, s, H-19), 1.14 (3H, s, H-18);^13^C-NMR (126 MHz, C_5_D_5_N) *δ*: 215.63 (C-22), 213.70 (C-3), 213.33 (C-11), 170.60 (COCH_3_), 141.78 (C-5), 120.65 (C-6), 82.09 (C-25), 80.64 (C-20), 72.91 (C-2), 70.81 (C-16), 59.61 (C-17), 51.46 (C-14), 51.40 (C-4), 49.79 (C-13), 49.27 (C-9), 49.20 (C-12), 46.80 (C-15), 43.30 (C-8), 37.40 (C-1), 35.87 (C-24), 34.73 (C-10), 32.74 (C-23), 29.91 (C-28), 26.52 (C-27), 26.44 (C-26), 26.03 (C-21), 24.63 (C-7), 22.68 (COCH_3_), 22.31 (C-29), 20.85 (C-19), 20.49 (C-18), 19.45 (C-30)。以上数据与文献^[23]^报道基本一致,鉴定该化合物为23,24-二氢葫芦素B,化学结构式如图4d所示。

化合物5:白色针状结晶;[M+HCOO]^-^(*m/z*): 605.33,相对分子质量为560.33,推测分子式为C_32_H_48_O_8_;^1^H-NMR (500 MHz, C_5_D_5_N) *δ*: 5.96 (1H, m, H-6), 5.04 (1H, m, H-16), 4.28 (1H, s, H-3), 1.93 (3H, s, COCH_3_), 1.62 (3H, s, H-30), 1.53 (3H, s, H-21), 1.52 (3H, s, H-27), 1.51 (3H, s, H-26), 1.49 (3H, s, H-28), 1.24 (3H, s, H-29), 1.22 (3H, s, H-19), 1.10 (3H, s, H-18);^13^C-NMR (126 MHz, C_5_D_5_N) *δ*: 215.50 (C-22), 213.10 (C-11), 211.31 (C-2), 170.63 (COCH_3_), 139.80 (C-5), 121.96 (C-6), 82.08 (C-3), 81.47 (C-20), 80.58 (C-16), 70.76 (C-25), 59.40 (C-17), 51.38 (C-14), 49.69 (C-9), 49.25 (C-12), 49.06 (C-13), 47.34 (C-4), 46.76 (C-15), 43.53 (C-8), 40.26 (C-1), 37.01 (C-24), 35.87 (C-10), 32.64 (C-23), 26.53 (C-27), 26.44 (C-26), 25.96 (C-21), 25.02 (C-29), 24.57 (C-7), 22.68 (C-28), 22.40 (COCH_3_), 20.86 (C-30), 20.43 (C-18), 19.34 (C-19)。以上数据与文献^[24]^报道基本一致,鉴定该化合物为二氢异葫芦素B-25乙酸酯,化学结构如图4e所示。

## 3 结论

利用液-液萃取与HSCCC对罗汉果根中葫芦素类化合物进行分离纯化,一次进样即可得到5种高纯度的葫芦素类化合物,分别为葫芦素Q1、23,24-二氢葫芦素F-25-乙酸酯、葫芦素B、23,24-二氢葫芦素B、二氢异葫芦素B-25乙酸酯。本方法简单快速,分离效果好,为分离葫芦素类化合物提供了新的高效制备方法,同时也为罗汉果的合理开发利用提供了技术支撑,具有较好的应用价值。
